# Hypoglossal nerve palsy after gasless trans-axillary endoscopic thyroidectomy: a case report

**DOI:** 10.1186/s12893-021-01114-5

**Published:** 2021-03-09

**Authors:** Qiao-Fei Liu, Zhe-Wei Zhao, Ming Cui, Sen Yang, Quan Liao

**Affiliations:** Department of General Surgery, Peking Union Medical College Hospital, Peking Union Medical College, Chinese Academy of Medical Science, 1# Shuai Fu Yuan, Dong Cheng District, Beijing, 100730 China

**Keywords:** Complication, Hypoglossal nerve palsy, Gasless trans-axillary endoscopic thyroidectomy

## Abstract

**Background:**

Gasless trans-axillary endoscopic thyroidectomy (GTAET) has satisfactory cosmetic effects for the patients who have benign goiter and small thyroid carcinoma, however the complications of this surgical procedure have not been fully documented. Ipsilateral hypoglossal nerve palsy (IHNP) associated with GTAET has never been reported before.

**Case presentation:**

A 33-year old male patient presented with a 4 × 5 mm solid thyroid nodule in the right lobe. Papillary thyroid carcinoma was confirmed by the fine needle aspiration. He had strong cosmetic demand, therefore GTAET for right lobectomy and central cervical lymphadenectomy was performed in a supine position with cervical extension. Six hours after the operation, he developed tongue deviation to the right side, speech and swallowing difficulties, indicating IHNP. Head and cervical MRI showed no abnormality. The intravenous steroid was used for three days, and oral vitamin B1 and mecobalamin was prescribed for 1 month. Nine days after surgery, he was discharged. Three months after the operation, all the symptoms were completely resolved.

**Conclusions:**

To the best of the authors’ knowledge, this is the first case of IHNP after GTAET, which will be valuable to add our knowledge to diagnose and treat rare complications of GTAET.

## Background

Cervical scar after thyroidectomy is a significant psychosocial burden for patients. Compared to the traditional cervical routine, minimally invasive and cervical scar-less thyroidectomy has gained in popularity [1]. Varieties of remote routine have been developed during the last 20 years, including anterior chest approach, trans-axillary approach, trans-oral approach, and retro-auricular approach, by using endoscopic or robotic instruments with or without gas insufflation [2]. Although each of these approaches has its advantages and drawbacks for certain subpopulations of patients, all of them bring satisfactory cosmetic outcomes without a cervical scar. Gasless trans-axillary endoscopic thyroidectomy (GTAET) is a newly developed surgical procedure, which is especially suitable for hemithyroidectomy [3]. Although iatrogenic injury of the parathyroid gland, recurrent laryngeal nerve, trachea, esophagus, and cervical vessels have been reported, GTAET is regarded as a safe surgical procedure with a low incidence of complications in general [4–6]. To the best of the authors’ knowledge, this is the first reported case of ipsilateral hypoglossal nerve palsy (IHNP) associated with GTAET.

## Case presentation

The patient was a 33-year old muscular man. He had a history of thyroid nodule in the right lobe for 2 years before visiting our hospital. Ultrasound showed a 4 × 5 mm hypoechogenic solid mass in the right lobe with irregular shape and unclear margin. The fine needle aspiration confirmed it was a papillary thyroid carcinoma. The thyroid function was normal. He had no other significant medical history or familial history. He was allergic to penicillin. The preoperative laryngoscopy examination showed no abnormality of vocal cords. He was an official of the government and had a strong willing to receive cosmetic surgery. Therefore, GTAET for right hemithyroidectomy and central cervical lymphadenectomy was performed. The paraffin pathological examination confirmed it was papillary thyroid carcinoma without lymph node metastasis.

**Fig. 1:**
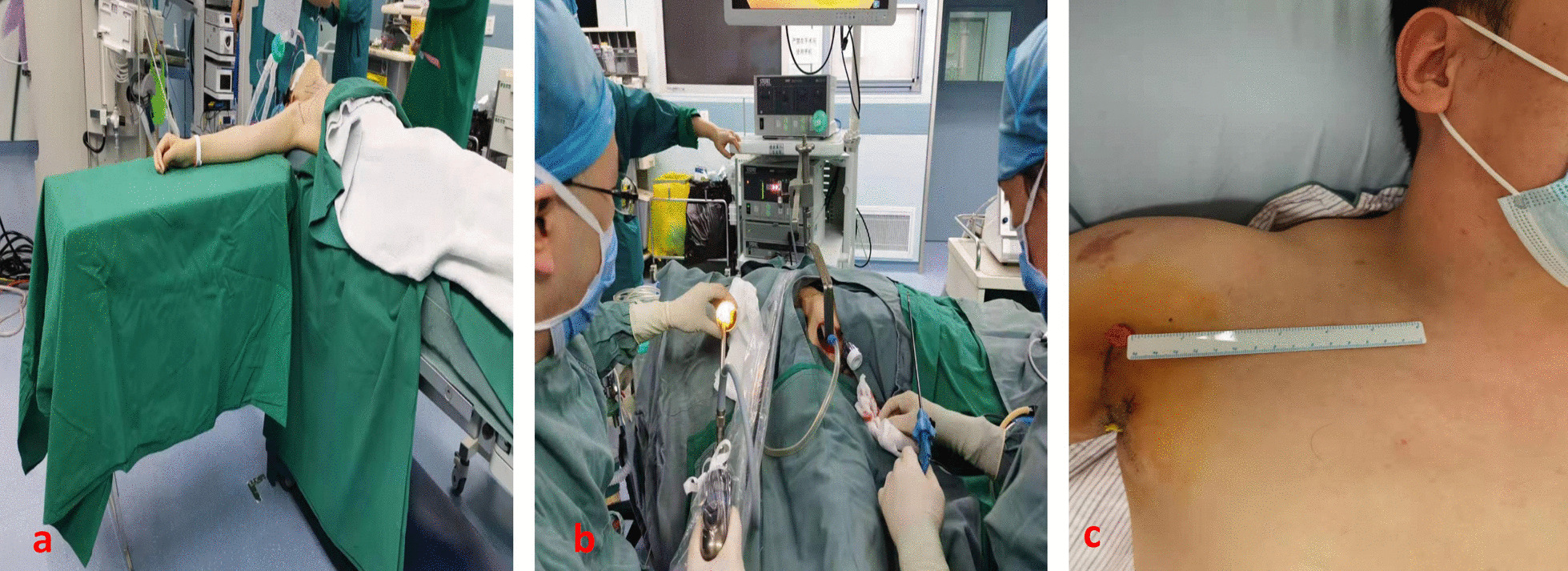
**a** surgical position of the patient; **b** out-side review of the surgical field; **c** surgical incision, 3 days after surgery

In brief, the GTAET was performed under general anesthesia using an endotracheal tube. The supine position with cervical extension was adopted. The patient’s right arm was abducted and placed on a bench. A 5-cm skin incision was made in the axillary fossa, and the skin flap was elevated. A 5-mm trocar was inserted inferior to the axillary incision. A 30-degree endoscope was placed through the axillary incision. The sternocleidomastoid muscle (SCM) and strap muscles were mobilized and elevated by a retractor. The carotid sheath was exposed and omohyoid muscle was cut. The intraoperative neural monitoring (IONM) was applied and no muscle relaxant was used to get better feedback of electronic stimulation. The recurrent laryngeal nerve was exposed and protected well. The isthmus was divided, and the right lobectomy with central neck dissection was completed. A suction drain was inserted, and the wound was closed. The signal of IONM of the recurrent laryngeal nerve was normal (Fig. 1). The patient was a muscular man, and the SCM and strap muscles were very strong, it was very difficult to get a satisfactory surgical field. It took us 3.5 h to finish this operation, and there was a total of 20 ml blood loss.

**Fig. 2:**
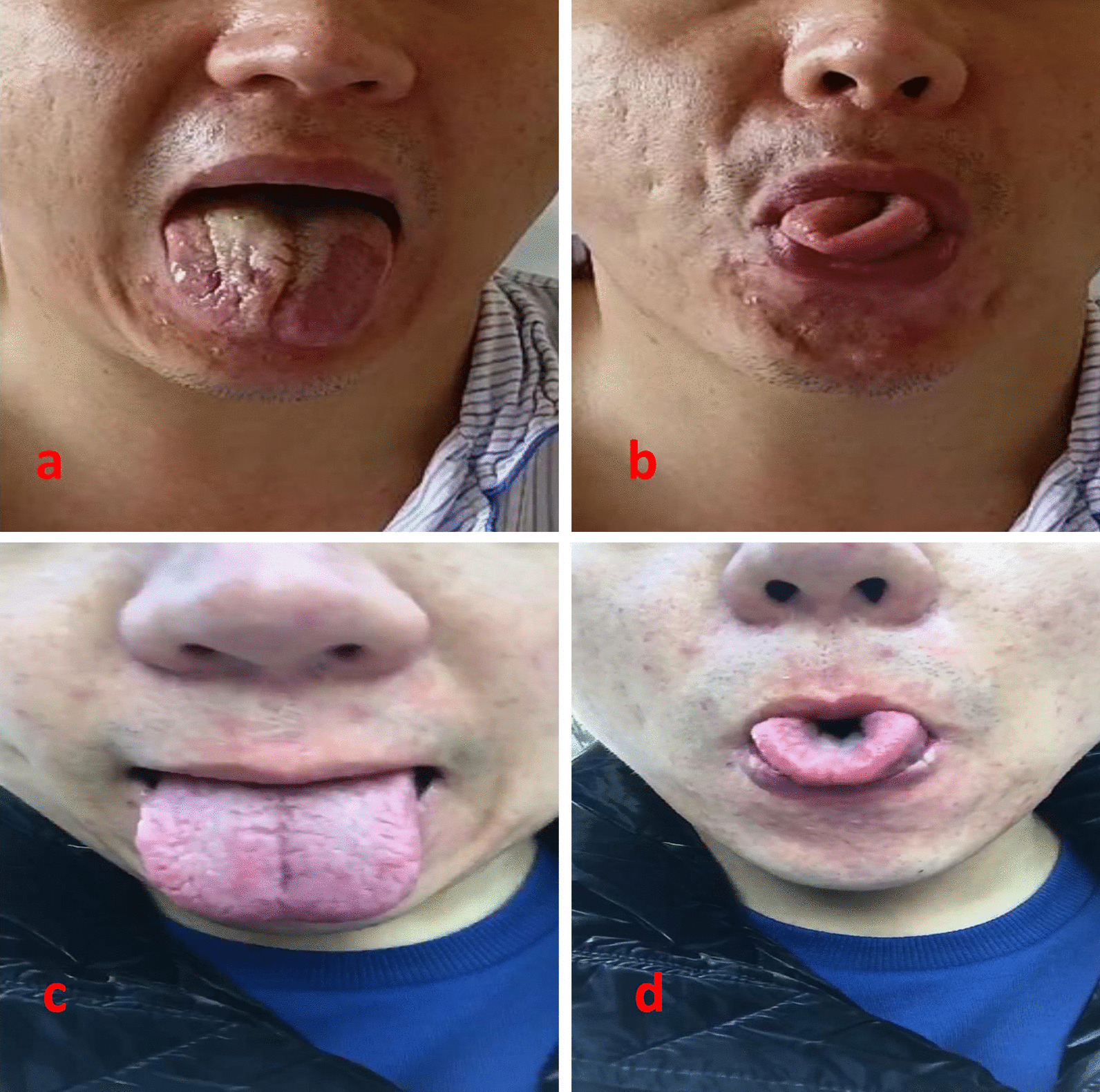
**a** flatted tongue deviated to the right side, 6 h after surgery; **b** rolled tongue deviated to the right side, 6 h after surgery; **c** flatted tongue in the middle-line, 3 months after surgery; **d** rolled tongue in the middle-line, 3 months after surgery

Six hours after surgery, he complained of tongue deviation to the right side, speech disturbance, and swallowing difficulties. Following the advice of the consultant neurologist, an MRI scan was performed, and the results showed no abnormality. An iatrogenic IHNP was confirmed by the consultation of neurologist. Intravenous 100 mg hydrocortisone was used twice per day for 3 days and oral vitamin B1 and mecobalamin were prescribed. He was discharged 9 days after surgery and oral vitamin B1 and mecobalamin were continued for 3 weeks. Three months after surgery, all the symptoms completely disappeared (Fig. 2).

## Discussion and conclusion

Most of the cases of iatrogenic hypoglossal nerve injury occur after direct transection or ligation of the hypoglossal nerve [7]. Indirect injury of the hypoglossal nerve is a rare finding, and there are only several case reports following laryngoscopy, laryngeal mask airway, transoral intubation, and poor body position [8]. After searching the public database, including PubMed, Web of Science, and Scopus with terms “hypoglossal nerve” and “thyroidectomy”, there were only two case reports. Bshait et al. [9]reported an IHNP following an open left hemithyroidectomy with cervical incision which occurred 3 h after surgery and a two-day course of corticosteroid was used. All the symptoms disappeared 3 months after the surgery. Ahn et al. [10]reported a case of IHNP after endoscopic thyroidectomy using a robotic system via cervical incision. After 6 months of following up, difficulties of speech and swallowing slightly improved, however the tongue deviation sustained without improvement. In a retrospective study of 5000 cases of trans-axillary endoscopic thyroidectomy, no HNP was reported. Herein, this is the first case of IHNP after GTAET [3].

Anatomically, hypoglossal nerve is beyond the surgical field of thyroidectomy and central cervical resection, which means iatrogenic hypoglossal nerve injury should be caused by indirect etiologies. A few etiologies may contribute to this rare complication. An MRI scan was performed to rule out the intracranial lesions and the result showed that there were no obvious abnormalities. Anesthetic procedures may lead to compression or laceration of hypoglossal never fibers, including cervical hyperextension, forceful placement of the laryngoscope on the lateral tongue base and cricoid pressure. Shah et al. reviewed 69 cases of HNP after general anesthesia with endotracheal tube placement. Otolaryngology was the predominant surgical specialty and the majority of the cases were male. These results indicated that larger dimensions of the hyoid bone, surgical position, and increased endotracheal mask airway cuff pressure were the risk factors for HNP [8]. In a series analysis of 234 cases of HNP, the male was also found to be an independent risk factor [7]. Distant stretching, laceration, compression of surgical procedure also may lead to HNP. This patient was in a supine position with the head turned to the opposite side. He was a muscular young man with strong SCM and strap muscles. To obtain a better surgical view, the SCM and strap muscles were persistently retracted for nearly 3 h. Due to the use of IONM, no muscle relaxant was used, which may exacerbate the resistance of SCM and strap muscles. The forceful pull of the superior polar of thyroid may also stretch the surrounding tissue of the hypoglossal nerve. All these procedures may lead to compression or laceration of the hypoglossal nerve. However, it is extremely difficult to give a definitive etiology of this rare case.

Although there is no solid evidence to support that steroid is effective, we still used intravenous steroid for 3 days. As well, oral vitamin B1 and mecobalamin were used for one month. In a retrospective analysis of 69 cases of HNP after general anesthesia, 80% of the patients recovered well [8]. Although this patient suffered an extremely rare complication, he recovered well and all the symptoms disappeared 3 months after surgery. He did not show any complaint and was satisfied with the treatments. We are still following up the patient to observe the HNP will reoccur or not.

In conclusion, HNP substantially affects the functions of speaking, biting, and swallowing, which is a significant psychosocial and physical burden for the patients. HNP could be permanent and no effective drug is available. Therefore, iatrogenic HNP should be prevented as much as possible. GTAET a generally safe surgical procedure that has substantial cosmetic effects, and IHNP is an unreported complication of GTAET before, and this first reported cases will add our knowledge and experience to diagnose and treat this rare complication [11]. Although no definitive etiologies were found, male patient, strong SCM and strap muscle, cervical hyperextension, forceful surgical and anesthetic manipulations may be the risk factors for iatrogenic HNP, which should be taken into account before performing a GTAET.

## Data Availability

All data and materials are available when requested to the corresponding author (Prof. Liao, lqpumc@126.com).

## Abbreviations

IHNP: Ipsilateral Hypoglossal nerve palsy
IONM: Intraoperative neural monitoring
GTAET: Gasless transaxillary endoscopic thyroidectomy
SCM: Sternocleidomastoid muscle

## References

[1] Berber E, Bernet V, Fahey TJ (2016). American thyroid association statement on remote-access thyroid surgery. Thyroid.

[2] Tae K, Ji YB, Song CM (2019). Robotic and endoscopic thyroid surgery: evolution and advances. Clin Exp Otorhinolaryngol.

[3] Kim MJ, Nam KH, Lee SG (2018). Yonsei experience of 5000 gasless transaxillary robotic thyroidectomies. World J Surg.

[4] Jantharapattana K, Maethasith J (2017). Transaxillary gasless endoscopic thyroidectomy versus conventional open thyroidectomy: a randomized study. Eur Arch Otorhinolaryngol.

[5] Stang MT, Yip L, Wharry L (2018). Gasless transaxillary endoscopic thyroidectomy with robotic assistance: a high-volume experience in North America. Thyroid.

[6] Hakim Darail NA, Lee SH, Kang SW (2014). Gasless transaxillary endoscopic thyroidectomy: a decade on. Surg Laparosc Endosc Percutan Tech.

[7] Stino AM, Smith BE, Temkit M (2016). Hypoglossal nerve palsy: 245 cases. Muscle Nerve.

[8] Shah AC, Barnes C, Spiekerman CF (2015). Hypoglossal nerve palsy after airway management for general anesthesia: an analysis of 69 patients. Anesth Analg.

[9] Bu Bshait M, Alyami H, Al-Osail E (2018). Ipsilateral hypoglossal nerve palsy following left hemithyroidectomy: case report and review of literature. Int J Surg Case Rep.

[10] Ahn SW, Kang KH (2015). Hypoglossal nerve palsy following the robotic thyroidectomy for the papillary thyroid carcinoma: a case report. Int J Surg Case Rep.

[11] Aidan P, Bechara M (2017). Gasless trans-axillary robotic thyroidectomy: the introduction and principle. Gland Surg.

